# Characterization of *Burkholderia pseudomallei* Strains Using a Murine Intraperitoneal Infection Model and *In Vitro* Macrophage Assays

**DOI:** 10.1371/journal.pone.0124667

**Published:** 2015-04-24

**Authors:** Susan L. Welkos, Christopher P. Klimko, Steven J. Kern, Jeremy J. Bearss, Joel A. Bozue, Robert C. Bernhards, Sylvia R. Trevino, David M. Waag, Kei Amemiya, Patricia L. Worsham, Christopher K. Cote

**Affiliations:** 1 Bacteriology Division, United States Army Medical Research Institute of Infectious Diseases (USAMRIID), Fort Detrick, Frederick, Maryland, United States of America; 2 Biostatisitics Division, United States Army Medical Research Institute of Infectious Diseases (USAMRIID), Fort Detrick, Frederick, Maryland, United States of America; 3 Veterinary Pathology Division, United States Army Medical Research Institute of Infectious Diseases (USAMRIID), Fort Detrick, Frederick, Maryland, United States of America; Tulane University School of Medicine, UNITED STATES

## Abstract

*Burkholderia pseudomallei*, the etiologic agent of melioidosis, is a gram-negative facultative intracellular bacterium. This bacterium is endemic in Southeast Asia and Northern Australia and can infect humans and animals by several routes. It has also been estimated to present a considerable risk as a potential biothreat agent. There are currently no effective vaccines for *B*. *pseudomallei*, and antibiotic treatment can be hampered by nonspecific symptomology, the high incidence of naturally occurring antibiotic resistant strains, and disease chronicity. Accordingly, there is a concerted effort to better characterize *B*. *pseudomallei* and its associated disease. Before novel vaccines and therapeutics can be tested *in vivo*, a well characterized animal model is essential. Previous work has indicated that mice may be a useful animal model. In order to develop standardized animal models of melioidosis, different strains of bacteria must be isolated, propagated, and characterized. Using a murine intraperitoneal (IP) infection model, we tested the virulence of 11 *B*. *pseudomallei* strains. The IP route offers a reproducible way to rank virulence that can be readily reproduced by other laboratories. This infection route is also useful in distinguishing significant differences in strain virulence that may be masked by the exquisite susceptibility associated with other routes of infection (e.g., inhalational). Additionally, there were several pathologic lesions observed in mice following IP infection. These included varisized abscesses in the spleen, liver, and haired skin. This model indicated that commonly used laboratory strains of *B*. *pseudomallei* (i.e., K96243 and 1026b) were significantly less virulent as compared to more recently acquired clinical isolates. Additionally, we characterized in *vitro* strain-associated differences in virulence for macrophages and described a potential inverse relationship between virulence in the IP mouse model of some strains and in the macrophage phagocytosis assay. Strains which were more virulent for mice (e.g., HBPU10304a) were often less virulent in the macrophage assays, as determined by several parameters such as intracellular bacterial replication and host cell cytotoxicity.

## Introduction

*Burkholderia pseudomallei*, the causative agent of melioidosis, is a gram-negative, facultative intracellular pathogen and has the ability to survive and replicate in both phagocytic and nonphagocytic cells [1–3]. Melioidosis cases are most commonly reported from countries located in Southeast Asia and the Oceania regions, with the greatest number of cases in Thailand, Vietnam, Cambodia, Laos, Malaysia, Singapore, and Northern Australia [4–9]. The disease has also been observed in the South Pacific, Africa, India, and the Middle East. In addition, sporadic cases of melioidosis have occurred in tropical locations in the western hemisphere [3,10–13]. Melioidosis cases that occur in non-endemic regions are normally associated with time spent in endemic areas, and there has been recent evidence that imported animals can harbor this bacterium [14,15]. The routes of infection include percutaneous inoculation, inhalation, or ingestion of contaminated food or water. Seasonal monsoonal rainfall is suspected to increase aerosolization of the bacteria resulting in pneumonia [7,16–18].

The incubation period varies depending upon the dose, route of infection, *B*. *pseudomallei* strain characteristics, and host predisposition. The incubation period can range from 1–21 days (mean of 9 days) [19]. However, individuals with risk factors such as diabetes, alcoholism, cirrhosis, thalassemia, lung disease, or other immunosuppressive conditions are considerably more likely to develop symptomatic infections [17,20]. Melioidosis normally presents as a febrile illness, however, this disease has an incredibly diverse list of clinical presentations including acute localized soft tissue infections, acute pulmonary infections, septicemia, and chronic localized infections [3,4]. Clinical disease with *B*. *pseudomallei* is generally caused by hematogenous spread of bacteria and seeding to various organs within the host. Although acute infections in individuals with predisposing risk factors are most common, latent infections with or without acute reactivation long after initial exposure also occur with melioidosis [19,21]. It has been postulated that after the initial phase of infection, *B*. *pseudomallei* can persist in a dormant stage in macrophages for months or years.

Intracellular survival and cell-to-cell spread may provide *B*. *pseudomallei* protection from the humoral immune response [1] and likely contributes to the need for a prolonged course of carefully selected and administered antibiotics. *B*. *pseudomallei* continues to be a public health concern in endemic areas and for military personnel serving in these regions because of its potential long latency period and ability to evade the host immune response [22–24]. Additionally, *B*. *pseudomallei* has been designated a Tier 1 agent due to its potential use as a biological agent which could represent a serious threat to public health and safety [2,25]. It is for these reasons that effective therapeutics and vaccines are needed.

A well characterized animal model must be used to generate both efficacy and safety data for such novel medical countermeasures and potentially support future pre-clinical studies. In this report, we discuss both *in vitro* characterization and *in vivo* virulence determination of a panel of 11 strains of *B*. *pseudomallei*. Our *in vitro* characterization included growth curves, LPS profiles, and macrophage phagocytosis/cytotoxicity assays. We described virulence as measured by median lethal dose (LD_50_) determinations using the intraperitoneal (IP) murine model of infection. We chose to pursue the IP infection route, in part, because it offers an easy model that can be reproduced by many laboratories. Additionally, we highlighted several noteworthy pathologies associated with administering *B*. *pseudomallei* to mice via the IP route.

## Materials and Methods

### Strain characterization

The 11 strains of *B*. *pseudomallei* used in this study were selected by collaborating U.S. federal agencies: Defense Threat Reduction Agency (DTRA) and Biomedical Advanced Research and Development Authority (BARDA). These strains represent an index set of strains to be used in subsequent vaccine and therapeutic countermeasure development research. The strains are all human isolates for which histories and passage information are available and represent both commonly used laboratory strains and relatively new clinical isolates, as described in S1 Table and elsewhere ([26] and Critical Reagents Program/USAMRIID Unified Culture Collection, unpublished). Master seed banks (MSB) of the strains were prepared by streaking the frozen sample received onto a sheep blood agar (SBA) plate and incubating at 35°C for 24 h. A single colony from the plate was used to inoculate a starter culture of calcium-adjusted Mueller Hinton II (CaMHB II) broth. This culture was grown at 37°C with shaking to an OD_600_ between 0.1 and 0.3 and diluted to an OD_600_ of 0.001 in fresh CaMHB II. This culture was incubated at 37°C for approximately 12 h until it reached mid-log growth phase. The resulting culture was mixed with an equal portion of CaMHB II supplemented with 12.5% glycerol (final concentration) and frozen in aliquots. A working stock bank (WSB) was prepared using 10 μl of the MSB to inoculate a fresh CaMHB II culture which was grown to mid-log phase, combined with an equal volume of CaMHB II with 25% glycerol, and distributed into cryovials (500 μl aliquots) for storage at -70°C.

### *In vitro* growth kinetics analyses

Growth curves were prepared as follows. Flasks containing 50 ml of glycerol tryptone broth (GTB, 1% tryptone, 0.5% NaCl, 4% glycerol) were each inoculated with approximately 200 μl of WSB material. The flasks were shaken at 200 rpm at 37°C. Aliquots were removed at selected times, and OD_620_ readings were measured using a spectrophotometer. Samples were periodically plated on SBA plates for determination of viable colony forming units (CFU).

### Lipopolysaccharide (LPS) phenotyping of *B*. *pseudomallei* strains

*B*. *pseudomallei* cells from each of the 11 strains were streaked onto SBA plates and incubated at 37°C for 48 h. From each plate, approximately 5 colonies were suspended in GTB and then incubated at 37°C with shaking at 200 rpm overnight. The cultures were then diluted with GTB to an OD_620_ of approximately 1.0 and were heat-killed at 90°C for 90 minutes. After sterility was confirmed, LPS extractions were performed with 5 ml of killed cells for each strain using the procedure described by Yi and Hackett [27]. The purified LPS was separated on 10–20% Tricine gels (Thermo Fisher Scientific, Waltham, MA). To help analyze the LPS profiles, we used a mouse-derived monoclonal antibody which was generated against inactivated *B*. *pseudomallei* strain 1026b (11G3-1). A western blot was performed with a 1:2000 dilution of 11G3-1 then a 1:5000 dilution of peroxidase labeled goat anti-mouse IgG secondary antibody was used (KPL, Gaithersburg, MD). The blot was developed using colorimetric detection with TMB Membrane Peroxidase Substrate (KPL). Alternatively, silver staining was conducted using the method described by Tsai and Frasch [28].

### Macrophage assays

*Burkholderia* strains were examined for their ability to infect macrophage-like cells and to induce cell damage using a protocol similar to that described in other studies [29–32]. The J774.A1 murine-derived macrophage-like cell line was cultured in Dulbecco’s Modified Eagle Medium (DMEM) with glucose (Lonza, Walkersville, MD), glutamine, and 10% fetal bovine serum in 24-well plates (in wells with or without coverslips). The cells were incubated for 2 days at 37°C with 5% CO_2_, at which time, growth to 90–95% confluence was achieved. On the day before the experiment, GTB was inoculated with bacterial growth from fresh SBA plate cultures of the bacteria and the flasks were incubated overnight at 37°C with shaking at 200 rpm. The densities of the overnight cultures were measured spectrophotometrically, suspensions adjusted to an OD_620_ of 1.0 (approximately 1 x 10^9^ CFU/ml) were prepared, and the macrophages were inoculated with the *Burkholderia* strains at a multiplicity of infection (MOI) of 10–20 CFU per macrophage (MOI for each experiment is listed in figure legend). The inocula were diluted and plated on SBA plates to determine the actual MOI. The infected cultures were incubated 1 h to allow uptake; and the cells were washed and incubated with kanamycin (250–500 μg/ml) for 2 h to kill residual extracellular bacteria. Infected cells were then washed, fresh medium was added, and cultures were reincubated for a total of 7–8 h. Infected cells were then processed for bacterial survival/replication and cytotoxicity. Lysates of infected macrophages collected after 1 h, 3 h, and 8 h (final time point) were diluted and plated in triplicate on SBA plates for viable count determinations. Samples were collected from each of three separate wells per strain. Cytotoxicity activity was assessed using several parameters: the extent of cell loss (% detached cells compared to uninfected controls), the proportion of dead cells by staining with live-dead dyes (trypan blue or propidium iodide-PI), overall morphologic changes by staining with histologic dyes (Diff-Quik), and an analysis of multi-nucleated giant cell (MNGC) formation. For the latter, the relative proportions of MNGCs compared to normal cells and of nuclear morphology types (multi-nuclear vs. single nucleus) were determined. For the cytotoxicity data, cells in a minimum of six separate fields (two for each of three Diff-Quik-stained coverslips) were counted to obtain the MNGC parameters and to estimate the extent of cell loss; three fields in duplicate trypan blue- or PI-stained wells were examined to determine the proportion of dead cells and estimate the percentage of cell loss. For all the cytotoxicity results, the values for the infected wells were normalized by comparing them to the counts of identically treated uninfected cell wells.

### Animal challenges: LD_50_ determinations in mice

Bacteria used for challenge were harvested from a late log phase culture grown in GTB medium at 37°C with shaking at 200 rpm. The bacteria were quantified via OD_620_ estimations and delivered IP in 200 μl of GTB. The delivered doses of bacteria were then verified by plate counts on SBA. Groups of 10 BALB/c mice (National Cancer Institute, Frederick, MD; female, 7–10 weeks of age at time of challenge) were challenged by the IP route with various doses of *B*. *pseudomallei*. Mice were monitored for clinical signs and symptoms for 60 days. Early endpoint euthanasia was employed in a uniform manner to limit pain and distress.

### Histological pathology

Post-mortem tissues were collected from several euthanized mice exhibiting noteworthy pathology and treated with 10% neutral buffered formalin for at least 21 days then subjected to histological analyses. Samples were embedded in paraffin and sectioned for hematoxylin and eosin (HE) staining, as previously described [33].

### Ethics statement

Animal research at the United States Army Medical Research Institute of Infectious Diseases (USAMRIID) was conducted under an animal use protocol approved by the USAMRIID Institutional Animal Care and Use Committee (IACUC) in compliance with the Animal Welfare Act, PHS Policy, and other Federal statutes and regulations relating to animals and experiments involving animals. The facility where this research was conducted is accredited by the Association for Assessment and Accreditation of Laboratory Animal Care International (AAALAC) and adheres to principles stated in the Guide for the Care and Use of Laboratory Animals (National Research Council, 2011). Challenged mice were observed at least daily for 60 days for clinical signs of illness. Humane endpoints were used during all studies, and mice were humanely euthanized when moribund, according to an endpoint score sheet. Animals were scored on a scale of 0–11: 0–2 = no significant clinical signs (e.g., slightly ruffled fur); 3–7 = significant clinical symptoms such as subdued behavior, hunched appearance, absence of grooming, hind limb issues of varying severity and/or pyogranulomatous swelling of varying severity (increased monitoring was warranted); 8–11 = distress. Those animals receiving a score of 8–11 were humanely euthanized by CO_2_ exposure using compressed CO_2_ gas followed by cervical dislocation. However, even with multiple observations per day, some animals died as a direct result of the infection.

### Statistical analyses

The differences in *in vitro* growth kinetics were evaluated statistically using GraphPad Prism, version 5.2 software (GraphPad Software, La Jolla, CA). Nonlinear regression analysis was done using the exponential model, as determined by curve fitting. The k parameter (rate constant) was used for best fit comparisons between the data sets for each strain. To estimate lethal dose response curves for each *B*. *pseudomallei* challenge strain, the following probit model was fit:

$$p\left(x_{ij}\right)\;=\;\Phi \left(\alpha_{i}+\beta_{i}\cdot \log_{10}Dose_{j}\right)$$

*i* indexes the challenge material, *j* indexes the dose levels of the challenge, and *α* and *β* are the slope and intercept, respectively. The number of non-surviving subjects was modeled as:

$$x_{j}∼\text{Binomial}\left(p,n_{j}\right)$$

The priors [34] for this model are:

$$\alpha_{i}∼\text{Cauchy}\left(0,\;10\right)\;\beta_{i}∼\text{Cauchy}\left(0,\;10\right)$$

Samples were drawn from the posterior distributions using Hamiltonian Monte Carlo [35] as implemented in Stan [36,37] using four chains each with a warmup of 5,000 draws followed by 12,500 samples for a total of 50,000 posterior points. Inversion of the probit model formula allowed for the construction of the posterior distributions of the median lethal dose (LD_50_) for each challenge material. This permitted the direct comparison of the posterior LD_50_ distributions of each pair of challenge materials which allowed probabilistic statements to be made regarding the likelihood that the LD_50_ of one challenge is smaller or larger than that of another challenge. All Bayesian estimates are presented with 95% highest posterior density (HPD) credible intervals. All Bayesian analyses were performed using Stan 2.1.0. All other statistics were performed using R 3.1.1.

## Results

### *In vitro* characterization of the *B*. *pseudomallei* strains

We performed *in vitro* characterization of the 11 *B*. *pseudomallei* strains to include growth kinetics and LPS profiling. To ensure that any differences observed during *in vivo* analyses were not attributable to a defect in growth, growth curves were constructed for all 11 strains based on the OD_620_ values recorded at intervals during incubation of the broth cultures (S1 Fig). The k parameter (rate constant) was used for best fit comparisons between data sets for each strain, and the resulting analysis revealed that the curves of all data sets were not statistically different (*P* = 0.1304).

**Fig 1:**
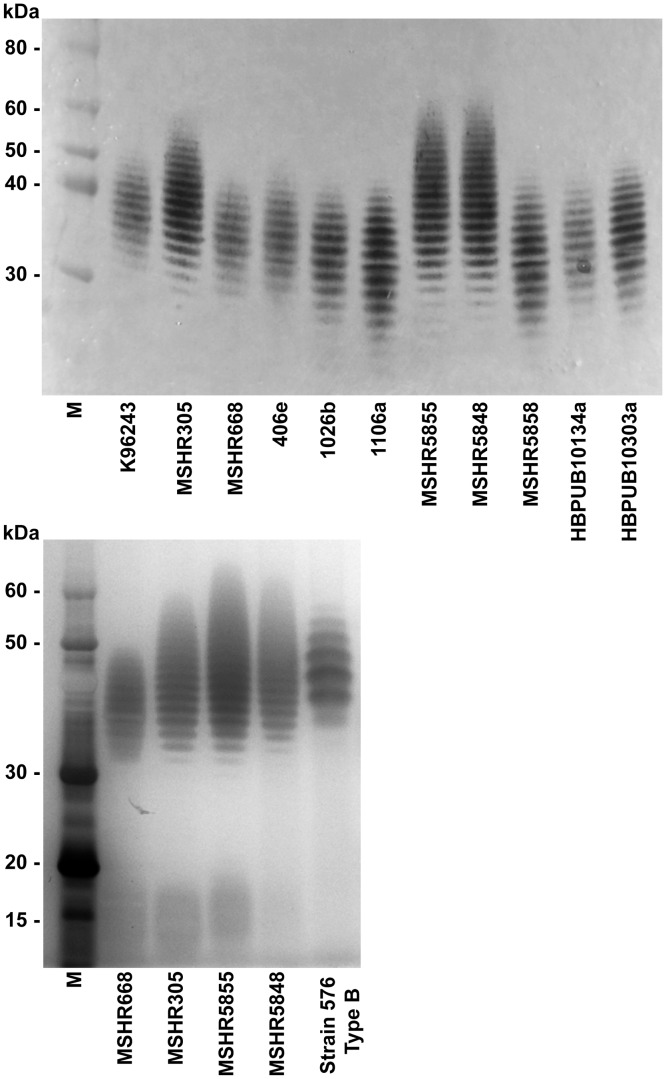
*B*. *pseudomallei* LPS profiles. **A.** Purified LPS samples from 11 *B*. *pseudomallei* strains were separated by SDS-PAGE. The gel was electroblotted, and the western blot was developed using a monoclonal antibody directed against *B*. *pseudomallei* LPS (11G3-1). **B.** Results were confirmed by silver staining. *B*. *pseudomallei* 576 displaying the LPS type B banding pattern is shown for comparison. M = molecular weight marker.

Additionally, we were interested in identifying any apparent variations in LPS profiles. The LPS banding profiles for the 11 *B*. *pseudomallei* strains were analyzed by SDS-PAGE and western blotting. The LPS phenotypes of all the strains appeared provisionally to be type A, and six of them (K96243, MSHR305, MSHR668, 406e, 1026b, and 1106a) have been reported previously to exhibit type A LPS [38,39]. However, a comparison of the molecular weight range of their LPS banding ladders suggested the presence of three distinct phenotypes (Fig 1). These may be type A variants or subtypes. MSHR5855 and MSHR5848 had identical LPS banding patterns with a range higher than any of the other strains (~26–60 kDa). MSHR305 displayed an intermediate range (~29–55 kDa), and the remaining strains exhibited the lowest LPS banding patterns, ranging from approximately 23–50 kDa, depending on LPS concentration. Results of the western blot in panel A were confirmed by silver stained gels, and *B*. *pseudomallei* 576 displaying the type B LPS banding pattern is shown for comparison (Fig 1B).

### Intraperitoneal mouse LD_50_ determinations

**Fig 2:**
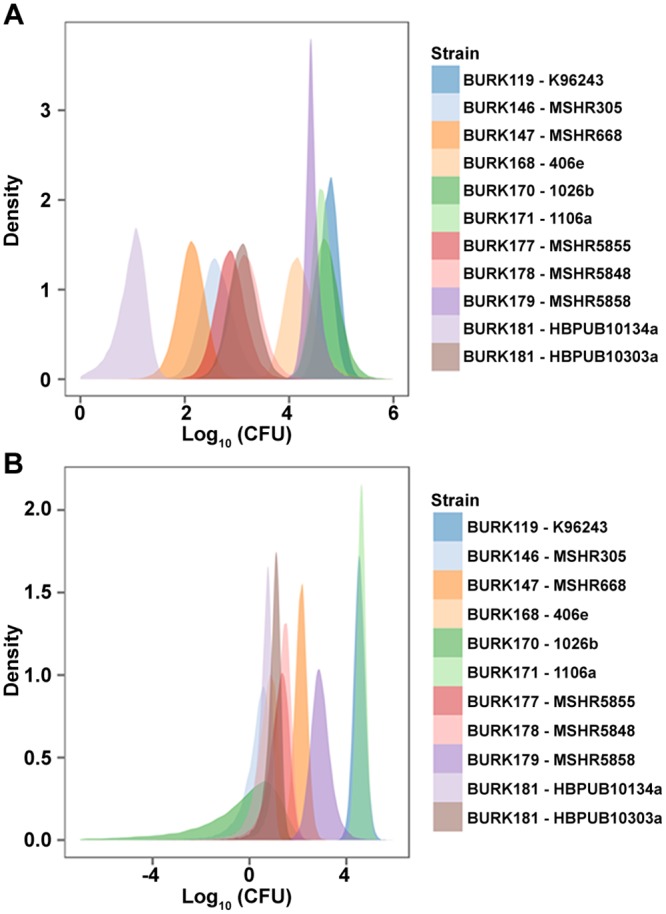
Statistical analyses of the LD_50_ determinations. Kernel density plot of the posterior distributions of the LD_**50**_ values of each *B*. *pseudomallei* isolate in BALB/c mice through **A.** 21 day survival challenge and **B.** 60 day survival challenge.

The LD_50_ values for each of the 11 *B*. *pseudomallei* strains were calculated at both 21 and 60 days after IP challenge (Table 1). Direct comparisons of the posterior samples of the LD_50_ values of each strain permit us to make probabilistic statements about how likely it is that one strain is more or less virulent than any other strain, given the observed data. Statistical analyses of day 21 data differentiated the 11 strains into three distinct virulence groups (Fig 2A), however, many of these differences were less significant when analyzing the data through day 60 (Fig 2B). The Bayesian analyses of the day 21 LD_50_ data (posterior distributions) indicated that the 11 strains could be divided into three major virulence (“potency”) groups composed of one, five, and five strains, respectively, as follows (from most to least virulent): HBPUB10134a (group 1); MSHR668 (group 2A) and MSHR305, MSHR5855, MSHR5848, and HBPUB10303a (group 2B); and K96243, 406e, 1106a, 1026b, and MSHR5858 (group 3). Strain MSHR668 was statistically more virulent than the other four strains in the intermediate virulence group, although these five strains appear to cluster similarly as illustrated in Table 1 and Fig 2. The day 60 LD_50_ data rankings again placed HBPUB10134a as most potent, K96243 and 1106a again as having LD_50_ values which were the largest and distinguishable from all others, and the remaining strains fell in the intermediate range.

**Table 1:** Summary of LD_50_ statistics for *B. pseudomallei* strains in BALB/c mice challenged by IP route.

	Day 21			Day 60		
	95% Confidence Interval			95% Confidence Interval		
Strain	Dose	Lower	Upper	Dose	Lower	Upper
K96243*	6.2x104	2.7x104	1.4x105	3.5x104	1.2x104	1.1x105
1026b	5.1x104	1.7x104	1.8x105	1	0	1.5x102
1106a*	4.2x104	1.7x104	9.6x104	4.1x104	1.7x104	9.4x104
MSHR5858	2.7x104	1.4x104	6.6x104	8.2x102	1.3x102	7.2x103
406e	1.6x104	4.4x103	8.4x104	6	1	32
MSHR5848	1.5x103	3.9x102	6.3x103	26	4	1.0x102
HBPUB10303a	1.3x103	3.8x102	4.5x103	11	2	32
MSHR5855	7.5x102	2.0x102	2.9x103	18	1	95
MSHR305	4.0x102	1.0x102	1.7x103	3	0	18
MSHR668*	1.3x102	37	4.5x102	1.4x102	37	4.5x102
HBPUB10134a	10	2	32	5	1	14

*data included in Challacombe et al. [98]

### Noteworthy pathology associated with the IP route of infection

While the main goal of these studies was to characterize virulence using the IP route of infection and subsequently calculating LD_50_ values for each strain, there were several interesting clinical and gross pathologic findings that were associated with the IP route of infection which warranted further investigation. The majority of these clinical signs were observed in mice regardless of the bacterial strain used. The dose of the challenge material seemed to dictate the corresponding pathology, as the higher doses resulted in acute disease and more rapid death compared to the mice receiving moderate to low doses of bacterial challenge material. Following early endpoint euthanasia, relevant tissues were collected from mice exhibiting these clinical abnormalities.

### Abscess/pyogranuloma formation

**Fig 3:**
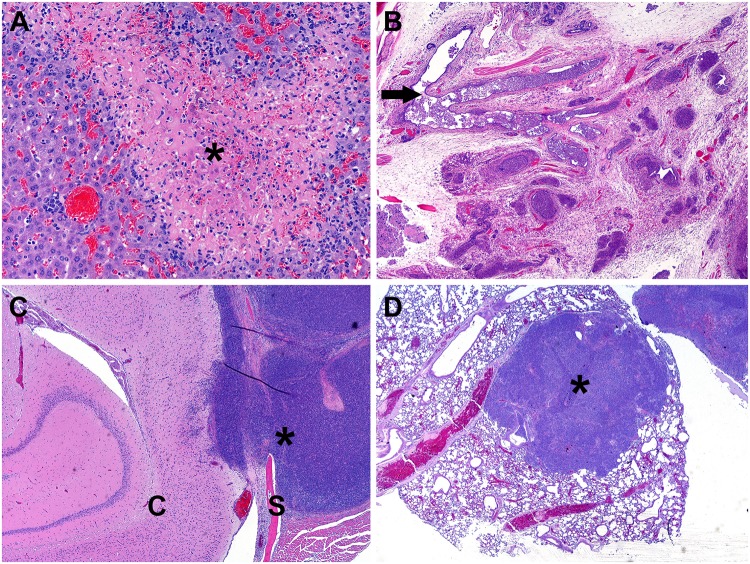
Representative histological lesions. **A.** Multifocal random areas of necrosis in the liver admixed with pyogranulomatous inflammation (BALB/c mouse challenged with 1.9 x 10^3^ CFU K96243, H&E 200X). The * identifies a necrotic area. **B.** Pyogranulomas and abscesses closely associated with lymphatic vessels within the haired skin which were markedly distended with and occasionally obliterated by large numbers of neutrophils (BALB/c mouse challenged with 1.9 x 10^3^ CFU K96243, H&E 40X). The arrow identifies the lumen of the lymphatic vessel containing inflammation. **C.** Pyogranulomatous inflammation (which originated from the haired skin and nasal sinuses) extending through the skull (S) into the meninges and cerebrum (C) (BALB/c mouse challenged with 1.4 x 10^4^ CFU K96243, H&E 40X). The * identifies an abscess. **D.** Significant area of the lung parenchyma obliterated by pyogranulomatous inflammation (BALB/c mouse challenged with 1.1 x 10^2^ CFU HBPUB10303a, H&E 20X). The * identifies an abscess.

**Fig 4:**
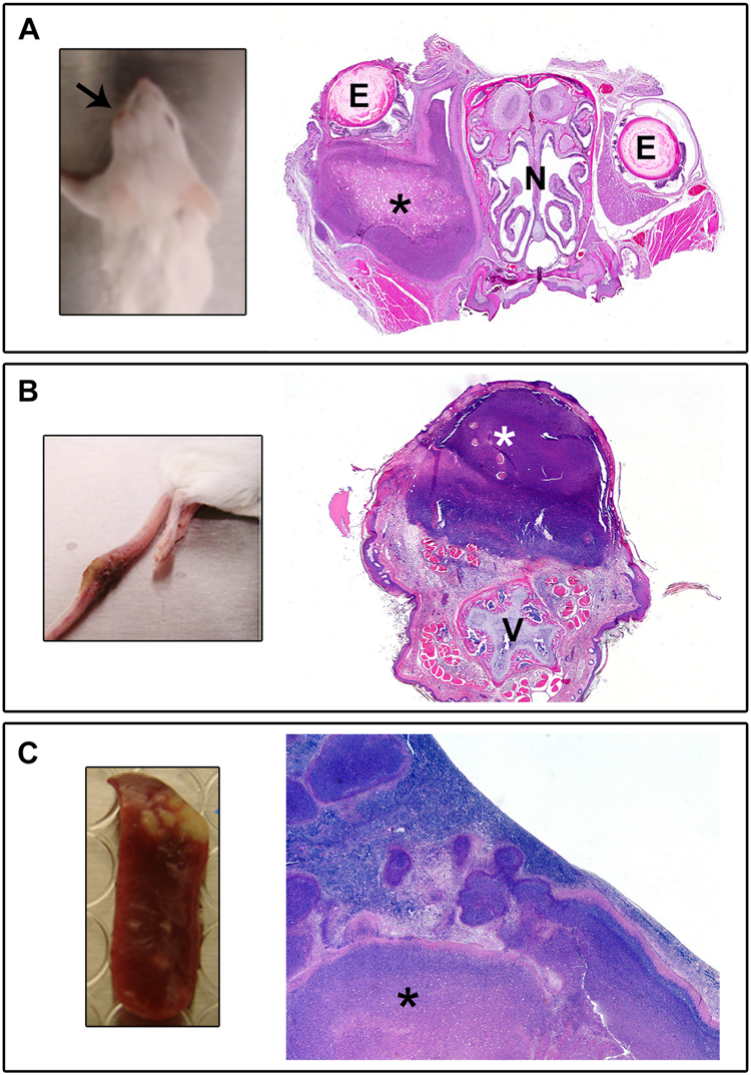
Several noteworthy clinical/gross observations and their histopathological correlates. **A.** Swelling of or around the eye (white arrow). Histologic evaluation revealed abscesses and pyogranulomatous inflammation filling the retro-orbital space causing the globe of the eye (E) to protrude from the socket (BALB/c mouse challenged with 4.2 x 10^4^ CFU 1106a, H&E 2X). The nasal cavity (N) is also marked for orientation. **B.** Large swellings on the tail (often seen in conjunction with hind limb swelling and/or paralysis). Histologic evaluation revealed pyogranulomatous inflammation from the haired skin of the tail extending into adjacent bone, skeletal muscle, and adipose tissue (BALB/c mouse challenged with 1.4 x 10^4^ CFU K96243, H&E 20X). The tail vertebra (V) is denoted for orientation. **C.** Firm white nodules in the spleen. Histologic evaluation revealed marked pyogranulomatous inflammation and necrosis (identified with an *) in the spleen which effaces normal white and red pulp (BALB/c mouse challenged with 9.0 x 10^2^ MSHR668, H&E 20X).

Mice were frequently observed with protuberances or swelling on various parts of the body. In some cases, small protrusions could be observed on the face and snout of the animals. In other cases, there were putative granulomas forming on the side or limbs of the animals. Upon histologic evaluation, it was confirmed that many of these visible protrusions could be attributed to abscess or pyogranuloma formation (Figs 3 and 4). A common site for abscess/pyogranuloma formation was the spleen, and pyogranulomatous inflammation could often be observed microscopically in the liver as well (Figs 3A and 4C). Pyogranulomatous inflammation in the lymphoreticular organs, particularly the spleen and liver, is typically seen in infections with *Burkholderia* species [40–45]. In some cases, the inflammation in the spleen was of sufficient severity and chronicity to cause fibrous adhesions between the spleen and adjacent organs, such as the pancreas, kidney, and ovary. Spleens were harvested from all mice that survived through day 60. The spleen weights often varied dramatically and were usually indicative of the overall bacterial burden associated with individual animals. Some spleens weighing up to approximately 3.0 g were observed (whereas normal spleens weigh between approximately 0.1 and 0.13 g), and they were somewhat difficult to remove from the peritoneum because of the fibrous adhesions. Finally, the lungs were often severely affected by this inflammatory response (Fig 3D), which may be indicative of a pneumonia that is secondary to a primary infection at a distant site. This type of secondary pneumonia has been associated with *B*. *pseudomallei* infections in humans [46].

Several of the mice had pathology involving one or both eyes. Eye issues were observed in mice infected with eight out of the 11 strains we examined. In some cases, the affected eye(s) appeared to be significantly swollen or protruding from the eye socket. Histological analysis revealed that the globe of the eye had only mild to moderate inflammation involving the cornea; however, the associated periorbital tissues, including extraocular muscles, optic nerve, lacrimal glands, and orbital bone were infiltrated and in some cases effaced by pyogranulomas or abscesses. These inflammatory cells often filled the retro-orbital space and caused the globe of the eye to protrude from the socket, resulting in the described gross appearance (Fig 4A). Similar inflammatory lesions were present throughout the coronal sections of the head in several mice. There have been documented cases of ocular melioidosis with similar descriptions [47,48]. Occasionally, these infections involved the inner and middle ear canals (which may be linked to the ear infections reported grossly in some animals) or extended through the sinuses into the meninges and cerebrum (Fig 3C).

### Hind limb paralysis

A more frequent observation was the significant impact to rear limb function, which was observed in mice infected with any of the 11 strains we examined. Clinically, the mice often showed an early “limp” in one rear leg, and this would progress to apparent paralysis in that leg. The first signs of single rear leg paralysis generally occurred between 6 and 30 days post-infection, depending upon the dose and strain of bacteria used. In some cases, the mice would develop double rear leg paralysis to varying degrees. There were occasional examples of mice developing double rear leg paralysis without an obvious single rear leg issue first. However, in the majority of the cases, double rear leg paralysis was observed between 1 and 11 days post onset of single rear leg issues. The observation of double rear leg paralysis was often a trigger for early endpoint euthanasia implementation. Upon pathological analyses, this hind limb involvement was associated with localized pyogranuloma formations. In several animals, significant pyogranulomatous inflammation was observed in the skin, skeletal muscle, bone, or peripheral nerves in the hind limbs which can be directly associated to the loss of motor function (paralysis) in the rear limbs. These inflammatory cells formed large pyogranulomas or abscesses often involving or associated with lymphatic vessels, which resulted in severe disruption of the aforementioned tissues (Fig 3B). Tail lesions were also observed, and although the affected region of the tail varied in severity, such lesions were a relatively common observation (Fig 4B).

### Macrophage infectivity and cytotoxicity of *B*. *pseudomallei*

An *in vitro* assay with murine cell line J774.A1 was used to compare the abilities of the *B*. *pseudomallei* strains to infect macrophages as a potential *in vitro* marker for evaluating bacterial infectivity. Several parameters were used to measure both quantitative bacterial viability and the extent of bacterial cytotoxic activity for the infected cells, as described in the methods.

**Fig 5:**
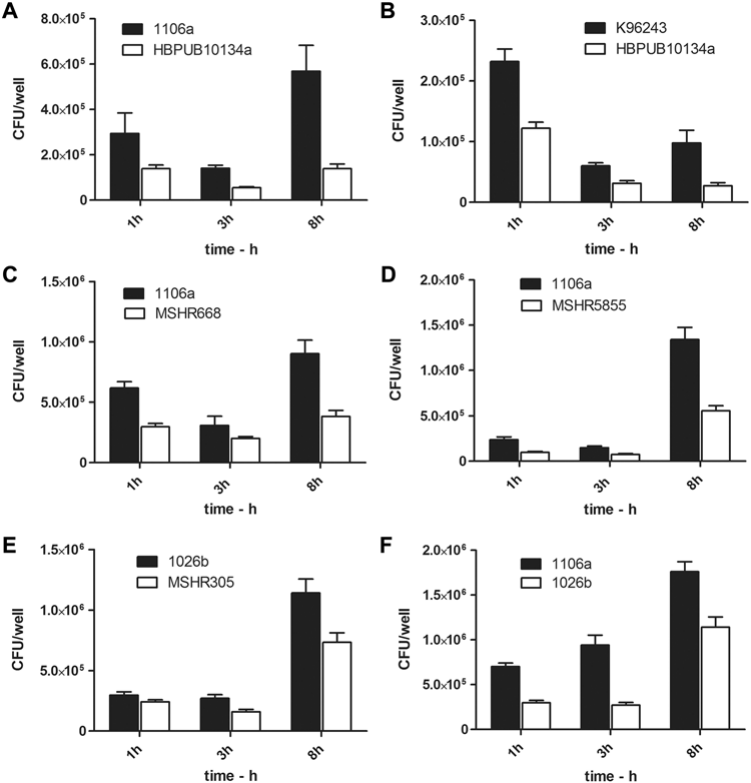
Comparisons of viable counts recovered from J774.A1 macrophages infected with different strains of *B*. *pseudomallei*. **A.** The MOIs for strains 1106a and HBPUB10134a were 17.2 and 13.7, respectively. Strain 1106a was phagocytosed to a greater extent than HBPUB10134a, as shown by the 1 h and 3 h viable counts and multiplied to a greater extent during the 5 h period between phagocytosis and the 8 h collections. The viable counts recovered from the macrophages of 1106a for all three time points were greater than those of HBPUB10134a: *P* = 0.0214 (1 h), *P* < 0.0001 (3 h), and *P* = 0.0018 (8 h). **B.** The MOIs for strains K96243 and HBPUB10134a were 27.5 and 26.5, respectively. The viable counts recovered from the macrophages of K96243 for all three time points were greater than those of HBPUB10134a: *P* < 0.0001 (1 h), 0.0006 (3 h), and 0.0052 (8 h). **C.** The MOIs for strains MSHR668 and 1106a were 8.9 and 5.8, respectively. The viable counts recovered from the macrophages of 1106a for all three time points were nearly or significantly greater than those of MSHR668: *P* < 0.0001 (1 h), *P* = 0.070 (3 h), and *P* = 0.0003 (8 h). **D.** The MOIs for strains 1106a and MSHR5855 were 12.4 and 7.7, respectively. The viable counts recovered from the macrophages of 1106a for all three time points were greater than those of MSHR5855: *P* = 0.0002 (1 h), 0.0019 (3 h), and < 0.0001 (8 h) **E.** The MOIs for strains 1026b and MSHR305 were 16.2 and 16.9, respectively. The viable counts recovered from the macrophages of 1026b were greater than those of MSHR5855 at the 3 h (*P* = 0.0015) and 8 h time points (*P* = 0.0097); the 1 h viable counts were not significantly different (*P* = 0.077). **F.** The MOI for both the 1026b and 1106a strains was 16.2. The viable counts recovered from the macrophages of 1106a for all three time points were greater than those of 1026b: *P* = 0.0001 for 1 h and 3 h time points, and *P* = 0.0029 for the 8 h time point.

**Fig 6:**
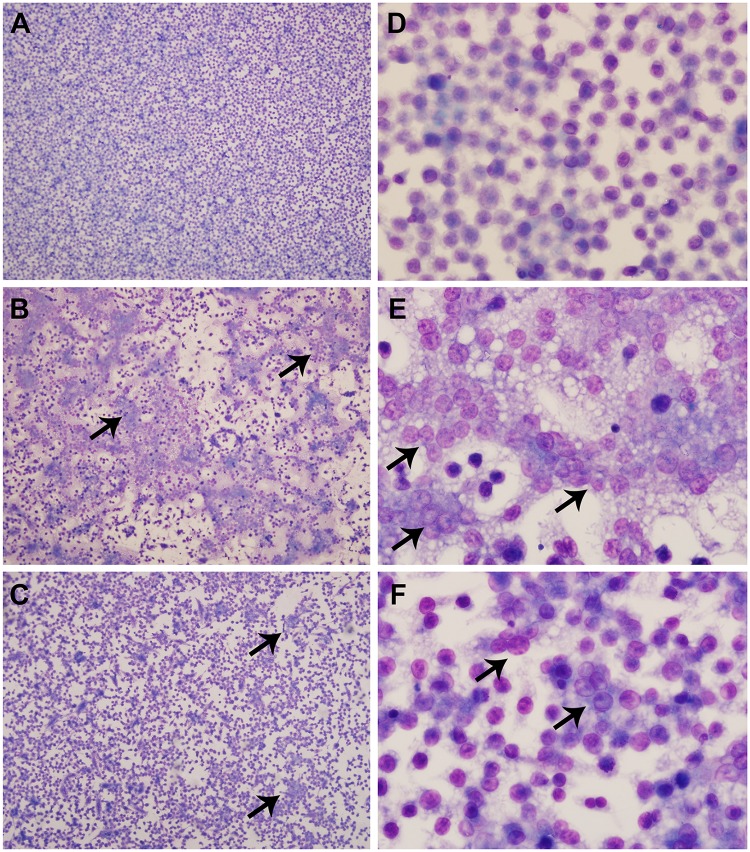
Comparative phenotypes of macrophages infected with strain 1106a or HBPUB10134a. Morphological changes in cells and in the extent of cell death/detachment as observed in Diff-Quik stains of cultures at the 8 h time point. The typical normal appearance of uninfected semi-confluent J774.A1 cells at low power, 100x **(A)** and high power, 600x **(D)**. **B, E:** Cells infected with 1106a, showing loss of monolayer and presence of numerous MNGCs and clusters of fused necrotic cells at 100x **(B)** and 600x **(E)**. **C, F:** Cells infected with HBPUB10134a and viewed at 100x **(C)** and 600x **(F)** demonstrated cell loss to a lesser extent than those with 1106a and promoted MNGC formation, albeit fewer in number and smaller in size. The arrows indicate examples of MNGCs.

As shown in Fig 5A and 5B. *pseudomallei* 1106a survived significantly better in macrophages than did *B*. *pseudomallei* HBPUB10134a. Strain 1106a was phagocytosed to a two-fold greater extent than HBPUB10134a, as shown by the 1 h (*P* = 0.0214) and 3 h (*P* < 0.0001) viable counts; and it multiplied to a greater extent during the 5 h period between phagocytosis and the 8 h collections (*P* = 0.0018). To account for differences in strain MOIs, the counts were normalized to the inoculum (CFU added at t_0_) or to the 3 h counts and expressed as percentages. These normalized values confirmed the differences, as shown in S2 Table. In addition to greater bacterial survival, 1106a was overall more cytotoxic for macrophages than HBPUB10134a, as shown by the greater percent cell loss from the wells, the larger proportion of MNGCs, and the higher mean number of nuclei within MNGCs (Table 2 and Fig 6). This *in vitro* virulence of 1106a is in contrast to the virulence in mice, as shown by the day 21 and day 60 IP LD_50_ values compared to those of HBPUB10134a (Table 1 and Fig 2). Differences between these strains in their extent of adherence and phagocytosis were examined further in a cytochalasin D (CD) treatment experiment. As shown in S2A Fig, the number of HBPUB10134a adherent to the macrophages (normalized as % of inoculum) was significantly less (*P* < 0.0001) than that of 1106a based on the counts from cells infected 1 h with CD-treated inocula. The extent of phagocytosis as determined by viable counts recovered after a further 2 h incubation of infected cells in kanamycin showed that HBPUB10134a was phagocytosed to an extent that was 11-fold less than that of 1106a, i.e., 0.05% and 0.55% (counts normalized as a percentage of the inoculum). Furthermore, the CFU recovered from CD- and kanamycin-treated 1106a-infected cells were significantly greater than those from HBPUB10134a-infected cells suggesting that fewer HBPUB10134a were phagocytosed in the presence of CD than 1106a (S2A Fig inset). To determine whether the reduced numbers of HBPUB10134a associated with the macrophages could be due to its more rapid exit or release from the cells, the CFU present in the media of cells infected and incubated in the absence of kanamycin were sampled. As shown in S2B Fig, the data indicated that there were fewer CFU of strain HBPUB10134a than strain 1106a present in the medium. Thus, these data suggest that the overall differences in survival of these strains in the macrophages might be attributed to different extents of adherence and phagocytosis, as well as to different extents of replication or killing during the post-phagocytosis incubation period (Fig 5A).

**Table 2:** Phenotypes of macrophages infected with *B*. *pseudomallei* strains—cytotoxicity comparisons.

						Mouse IP LD_50_	
Panel^a^	Strain	% cell loss^b^	% cells dead (TB)^b^	No. MNGC or necrotic cells (% of total)^c^	No. MNGC nuclei (% of total)^c^	day 21	day 60
A	1106a	50	15.0	8.3	45.7	4.2x10^4^	4.1x10^4^
	HBPUB10134a	15–20	≤1.0	2.4	9.2	10	5
B	K96243	40	8.3	5.6	25.7	6.2x10^4^	3.5x10^4^
	HBPUB10134a	25	4.1	2.3	8.8	10	5
C	1106a	60	61.8	12.0	73.4	4.2x10^4^	4.1x10^4^
	MSHR668	55	19.0	13.2	58.5	1.3x10^2^	1.4x10^2^
D	1106a	70–75	50.0	15.8	60.0	4.2x10^4^	4.1x10^4^
	MSHR5855	15	19.1	7.7	29.4	7.5x10^2^	18
E	1026b	55	12.5^b^	5.0	35.7	5.1x10^4^	1
	MSHR305	15–20	10.0	2.1	12.4	4.0x10^2^	3
F	1106a	≥85%	NA^b^	12.0	70.6	4.1x10^4^	4.1x10^4^
	1026b	55	10+	35.0	35.7	5.1x10^4^	1

^a^ Fig 6, panels A–F.

^b^Cytotoxicity is shown as the % cells lost from the macrophage layer, by trypan blue (TB) staining; and the % dead cells among those remaining by TB and/or PI staining. However, necrotic cells and advanced stage MNGCs with degraded nuclei are unstained by TB, and the values underestimate the actual proportion of dead cells.

^c^The values describe the approximate relative proportion of MNGCs among the total cells in six fields (%) with two from each of three replicate coverslips (600x). The major nuclear phenotypes, considering all the nuclei in the fields (typical large nuclei vs. nuclei present in MNGCs with intact stained nuclei or in necrotic fused cell masses with lightly stained nuclei), are expressed as percentages. Counts were done using Diff-Quik s

*B*. *pseudomallei* strain HBPUB10134a also demonstrated much less apparent infectivity and cytotoxicity for macrophages than *B*. *pseudomallei* K96243 (Fig 5B). K96243 was phagocytosed to a nearly two-fold greater extent than HBPUB10134a, as shown by the 1 h and 3 h macrophage lysate counts. It also multiplied significantly during the 5 h period between uptake and the 8 h collections, whereas HBPUB10134a viable counts were unchanged (Fig 5B and S2 Table). K96243 was more cytotoxic for macrophages, as shown by the more extensive cell detachment/loss, larger number of dead trypan blue-stained cells, larger proportion of MNGCs, and the higher mean number of nuclei within MNGCs (Table 2). The greater macrophage virulence of K96243 contrasts with its significantly higher day 21 IP LD_50_ for mice compared to HBPUB10134a (Table 1 and Fig 2). The macrophage infection comparison shown in Fig 5C (and S2 Table) follows the trend exhibited in the studies described in panels A and B. In this experiment, 1106a adhered to and was possibly phagocytosed by the macrophages to a greater extent than *B*. *pseudomallei* MSHR668, and multiplied more during the 5 h incubation period, as shown by the larger number of viable organisms recovered at the 8 h time point. In addition, 1106a induced even greater macrophage cytotoxicity than MSHR668 at 8 h, as evidenced by the greater percentage of trypan blue-stained cells and the presence of numerous notably large MNGCs, as well as fused, necrosing cells (Table 2). MSHR668 was significantly more virulent than 1106a, as determined by mouse IP LD_50_ values at days 21 and 60 post-exposure. Similar to the findings in Fig 5A and 5C, and as shown in Fig 5D and S2 Table, the viable counts recovered from macrophages infected with 1106a were greater than those of MSHR5855 for all three time points. MSHR5855 was much less cytotoxic than 1106a, as evidenced by the reduced cell killing and detachment and lesser proportion and size (number of nuclei) of MNGCs associated with infection by MSHR5855 (Table 2). As illustrated in Fig 5E, a comparison between strain 1026b and MSHR305 supported the trend observed in the other strain comparisons. The infection with 1026b produced greater numbers of viable counts at the 3 h and 8 h times but the 1 h recoveries of 1026b and MSHR305 were not significantly different. Also, 1026b was more cytotoxic, inducing more necrosis (as evidenced by trypan blue-positive and unstained necrotic MNGCs), greater cell detachment, and more extensive and larger MNGCs than MSHR305 (Table 2). However, MSHR305 was significantly more virulent than 1106a, as determined by mouse IP LD_50_ values at days 21 and 60 post-exposure.

A comparison performed with strains 1106a and 1026b demonstrated an interesting variation of the relationship described in panels A–E. Strain 1106a appeared to be phagocytosed to a greater extent than strain 1026b, and it was present in significantly higher concentrations at 8 h compared to 1026b (Fig 5F and S2 Table). Infection with strain 1106a also appeared to be associated with a greater extent of cytotoxicity and macrophage loss (S2 Table). Thus, 1106a appeared to survive, grow better, and exhibit more virulence for macrophages than 1026b. These differences in macrophage virulence with 1106a and 1026b were observed despite the fact that the strains had comparable day 21 IP LD_50_ values for BALB/c mice, although their day 60 IP LD_50_ values varied greatly (Table 1).

## Discussion

This work focused on characterizing a panel of 11 strains of *B*. *pseudomallei* for future use in animal model qualification and subsequent testing for medical countermeasures. We examined the growth kinetics of the strains and also determined their LPS profiles. We confirmed the LPS typing for several strains [39] and also showed that previously uncharacterized strains MSHR5858, HBPUB10134a, and HBPUB10303a appeared to be type A as well. Although strain MSHR305 was previously classified as type A [39], its LPS profile was consistently distinguishable from the other previously established type A strains under our test conditions. In addition, we tentatively identified strains MSHR5855 and MSHR5848 as type A because their ladder banding patterns were detected with the same monoclonal antibody (Fig 1A). However, their consistently higher band profiles again suggest that type A variants might exist among strains of the same known LPS phenotype. We will further investigate the strains that have potentially different banding patterns using genetic and structural analyses to determine whether they represent distinct subclasses of the known LPS types. Notably, the strains exhibiting these altered phenotypes in our studies were Australian isolates, and strains from Australia are known to display a higher diversity of LPS phenotypes [35]. It is also tempting to speculate about the location of the body from which these strains were isolated. For example, MSHR5855 and MSHR5848 were both isolated from sputum samples, and it is known that chronic cases of melioidosis have a higher proportion of patients exhibiting pneumonia [49]. Analogously, studies with isolates of the closely related *Burkholderia cepacia* complex from cystic fibrosis patients established that strains associated with chronic infection in the lungs frequently exhibited an altered LPS phenotype; rough instead of smooth in this case [50,51]. This suggests that *Burkholderia* LPS phenotypic changes could be associated with chronic infection. However, chronic bacterial infections are associated with a complicated network of virulence factors and regulatory mechanisms, as well as significant genomic alterations [52–54]. Accordingly, more work is required to fully understand the differences in *B*. *pseudomallei* LPS profiles and any potential effects on bacterial virulence or chronicity.

We chose the IP route of infection for these studies because this type of challenge can be readily performed. While aerosol infections are the most pertinent to the biodefense community, the IP route offered an accurate means to support initial characterization which would allow strain down-selection. Importantly, the use of BALB/c mice offered a model that could easily distinguish virulence attributes amongst these strains (Table 1). In some cases, mice are so sensitive to infections initiated by aerosolized bacteria that statistically significant strain differentiation based solely upon LD_50_ calculations may be very difficult ([55] and D. Waag, unpublished data). Our data demonstrate that the IP route of infection results in a prolonged infection and much higher LD_50_ values compared to those reported for inhalational routes of infection [55,56]. By using a route of infection that is less acute, it provides a means to potentially elucidate differences in bacterial strains which could be masked by using the inhalational route. The IP route also represents an important initial screening model useful for exploring pathogenesis and evaluating the efficacy of medical countermeasures prior to moving onto more difficult and costly models of inhalational disease [40,41,56–75].

This work also clearly demonstrates the importance of selecting appropriate study durations. Our data suggest that to accurately represent the disease initiated by IP inoculation, even 21 days of observation may not be sufficient. While observations made during the 14–21 day period in the BALB/c model may accurately reflect an acute stage of the disease, these observations do not necessarily reflect the overall health of the surviving animals and accordingly, the efficacy achieved when testing novel vaccines or therapeutics. As shown in this report, extended study durations will likely be required to demonstrate medical countermeasure efficacy in this (and likely any) animal model. It is important to note, however, that the LD_50_ values from the day 60 lethal dose analyses are more tenuous due to the greatly diminished pool of surviving mice.

The gross and histologic lesions seen in the IP challenged mice were consistent with those expected upon exposure to *B*. *pseudomallei*, and the reported clinical signs can be directly attributed to the numerous abscesses and pyogranulomatous inflammation seen histologically. Further studies are required to better understand if these clinical observations are more commonly associated with specific strains of bacteria, e.g., more virulent or less virulent *B*. *pseudomallei* strains, as determined in this model. However, these data document some relatively common clinical signs of BALB/c mice infected intraperitoneally with different *B*. *pseudomallei* strains, such as the pyogranulomatous inflammatory process occurring in multiple lymphoreticular locations resembling those described in other animals (mice, hamsters, NHPs, and humans [40,41,44,76]).

There have been several previous reports describing the susceptibility of BALB/c mice to *B*. *pseudomallei* infection by the IP route [58,70,73,77]. However, none of these reports used all of the strains described in the present work, the strain history provided was often brief, and clinical, pathological, and bacteriological characterization was minimal or not provided, with some exceptions. Chua et al. and Atkins et al. documented *in vivo* persistence of the inoculated *B*. *pseudomallei* strain in host organs up through 14 or 30 days, respectively [58,70]. Nevertheless, as reported here, the strains described previously exhibited a wide range of LD_50_ values, from approximately 3 to 5,650 CFU. Additionally, we determined LD_50_ values at a relatively early time post-challenge (day 21), as well as after an extended period of infection (day 60).

The *in vitro* macrophage phenotypes of several of the *B*. *pseudomallei* strains suggested the existence of a potential inverse association between a strain’s virulence in mice and its virulence in macrophages for at least a subset of *B*. *pseudomallei* strains. The macrophage infection phenotypes were assessed by determining both bacterial viability and macrophage cytotoxic activity. Fig 5 and S2 Table demonstrate the former, and Table 2 and Fig 6 illustrate the cytotoxicity parameters. As observed for other diverse *in vitro B*. *pseudomallei* phenotypes, there was some inter-experimental variability in the macrophage phenotypes, and the association was not precise for all strain comparisons. Nevertheless, the potential inverse association between mouse and macrophage virulence was observed in numerous assays and was especially clear-cut for comparisons of strains differing most in their day 21 and/or day 60 IP LD_50_ values (Table 1 and Fig 2). The comparisons which supported this observation involved seven strains (Fig 5); comparisons involving other strains, such as MSHR5848 and HBPUB10303a, also exhibited the inverse association.

In contrast, as described in the results, a comparison of the macrophage phenotypes of strains 1106a and 1026b revealed a potential variation of the possible inverse association between macrophage and mouse phenotypes (Fig 5F and Table 2 and S2 Table). Strain 1106a appeared to survive better in macrophages and induce greater cytotoxicity than 1026b, yet appeared to be comparably virulent for mice as indicated by their day 21 IP LD_50_ values. However, strains 1106a and 1026b differed significantly (> 4 logs) in their day 60 IP LD_50_ values (Table 1), as well as in aerosol virulence (D. Waag and C. Soffler, unpublished data). This differentiation in mouse virulence was reflected in the inverse macrophage infection parameters displayed by the two strains. This suggests that the macrophage phenotype might offer a marker of strain differences at later stages of infection. Nonetheless, the overall findings (Fig 5 and Table 2 and S2 Table) supported a potential inverse relationship between macrophage and mouse virulence for some strains of *B*. *pseudomallei*.

Interestingly, in studies with four strains of *B*. *thailandensis*, Wand et al. observed that the virulence of the strains in mice was potentially inversely associated with their macrophage cytotoxicity [78]. The two *B*. *thailandensis* strains that were most virulent in mice were much less cytotoxic for J774.A1 cells, as determined by lactate dehydrogenase (LDH) release and extent of MNGC formation, when compared to the other two strains that were highly attenuated in mice. Thus, these observations resemble those reported here for some *B*. *pseudomallei* strains. It should be noted that there are other possible explanations of our findings. For example, the reduced extent of macrophage infection by virulent strains, such as HBPUB10134a and MSHR5855, compared to lesser virulent strains, such as 1106a and K96243, might be due to faster egress of bacteria from macrophages (and killing by the antibiotic present in the medium) of the former compared to the latter strains instead of due to reduced intracellular viability. However, the consistent viable count data obtained at different time points (e.g., after removal of the antibiotic and further incubation of the infected cells) (Fig 5), the results of the CD treatment study (S2A Fig), and the quantitation of extracellular bacteria present in the medium of HBPUB10134a versus 1106a infected cells (S2B Fig) suggests that rapid release and dissemination may be a less likely explanation. However, alternate explanations for our observations, as well as the prospective studies described below are being pursued. Additionally, it is important to emphasize that we are not attempting to prove that there is a correlation or specific association between macrophage markers and the *in vivo* pathogenicity of, or host responses to, *B*. *pseudomallei*. Such an association requires more extensive natural history studies to validate the findings. It will also be necessary to demonstrate that an association between macrophage phenotypes and animal virulence can be reproduced in other murine cell lines and in primary cells, such as bronchial or bone marrow macrophages. Previous macrophage infection studies often used cell lines, the most common ones being the murine J774.A1 [31,32,79–83] and RAW264.7 [29,30,84–87] macrophage-like cells, as well as human monocyte-like cells, such as U937 [32,79,88]. We compared the J774.A1 and RAW264.7 cell lines in their responses to two strains of *B*. *pseudomallei* (MSHR668 and 1106a). The viable counts recovered of the strains were in similar relative proportions in both cell types, and the differences between MSHR668 and 1106a in their infection-associated cytopathology were observed in both J774.A1 and RAW264.7 cells. However, in our hands, the J774.A1 cells tended to produce a more homogeneous monolayer in the wells compared to RAW264.7 cells and thus, were more amenable to assessment of cytotoxicity. Specifically, several values (proportion of cells detached, % dead cells, proportion and size of MNGCs) were more difficult to quantitate in RAW264.7 than in J774.A1 cells due to the more heterogeneous distribution of the former in the wells.

Only after *in vitro* phenotypes, such as those provided by the macrophage phagocytosis assay, is thoroughly characterized and reproduced can the mechanisms underpinning their possible association with pathogenesis be investigated. For instance, a more attenuated and less destructive course of infection in the reticuloendothelial system may promote *in vivo* persistence and the evolution of infection to a more chronic form. Some of the strains that were the most virulent for mice (lowest 21 and 60 day LD_50_ values) conversely grew less aggressively in, and exhibited reduced cytotoxicity for, macrophages compared to strains that were more attenuated in the mice (Fig 5 and Tables 1 and 2 and S2 Table). Macrophages might provide a protected niche for these intracellular pathogens against host immune responses [89–91], and it could be speculated that it might be disadvantageous for the *Burkholderia* to destroy its host cell during certain stages in pathogenesis. Although these hypotheses are speculative, it seems clear that further investigations could reveal additional uses for macrophage and other *in vitro* cell assays in probing the mechanisms of *Burkholderia* pathogenesis.

It is generally recognized that *in vitro* assays of pathogen growth and survival in macrophages are in all likelihood suboptimal models of host infection and not necessarily predictive of *in vivo* differences in strain virulence [32,80,92,93]. It is certainly true that no relatively simple *in vitro* system can model the complex processes involved in pathogenesis. However, the results of this study suggest that taking advantage of several measurable aspects of the bacterial-macrophage interaction may reveal some consistent associations between a given stage in pathogenesis and a specific bacterial or host parameter(s) of the *in vitro* infection. Recently, an automated image acquisition and analysis method was developed and used to characterize the ability of *B*. *pseudomallei* to induce MNGC formation [85]. It is a quantitative and promising approach although it does not measure bacterial viability, and it may be more difficult to distinguish MNGCs from cell clumps by the imaging technique than by manual staining and microscopy. However, the technique may be of value in comparing related *B*. *pseudomallei* mutants differing in virulence. The quantitative image analysis results with a wild type strain and mutant derivatives agreed with the known mutant defects in ability to induce MNGCs. The assay was also used to identify small molecules that inhibit the MNGC process and thus might provide tools for probing the molecular targets for the MNGC phenotype. This work employed one strain, K96243, and derivatives of it; although the findings do not inform those of the present study, they exemplify the application of specific macrophage phenotype as a potential *in vitro* biomarker for infection. Macrophage modeling has been used in studies with *Burkholderia*, as well as other pathogens (e.g., *Yersinia pestis*, *Yersinia pseudotuberculosis*, *Francisella tularensis*, and *Bacillus anthracis*); to probe potential mechanisms of pathogenesis and host responses, such as putative roles in infection of specific bacterial-associated mechanisms and virulence factors [29–32,79–87,89,92–96]. Macrophage models have been essential in efforts to better understand the roles of the type III and type VI secretion systems (T3SS and T6SS) in the pathogenesis of disease, the mechanisms associated with specific factors encoded by these systems (facilitating intracellular survival, replication, movement, and spread to other cells), and the cellular responses employed by the host to contain these pathogens. For example, Kespichayawattana et al., used findings from a J774.A1 cell culture model to propose that the cell-to-cell spread of *B*. *pseudomallei* is mediated by its induction of actin-associated membrane protrusion and cell fusion [31]. Tandhavanant and Chantrati noted a partial association between colony morphotype switching of *B*. *pseudomallei* and survival fitness in J774.A1 and human cells, such as the U937 macrophage; however, significant strain variability was observed [32,80]. Using J774.A1 cells, Stevens and coworkers evaluated the activity of T3SS-encoded BopE and other T3SS-encoded proteins in cellular invasion and spread [82,83]. Burtnick et al. discovered a potential role of the type VI secretion system in survival of *B*. *mallei* and *B*. *pseudomallei* in animals and identified T6SS-encoded proteins which contributed to the ability of these pathogens to grow in RAW264.7 cells and induce MNGC formation [30,84]. Breitbach and coworkers and Utasincharoen et al. characterized the production of nitric oxide synthase and cytokines (interferons and tumor necrosis factor) by host cells in response to *B*. *pseudomallei* infection in RAW264.7 and primary bone marrow macrophages, respectively [86,87,97]. These and other reports demonstrate the importance of *in vitro* cell models in identifying potential mechanisms of bacterial pathogenesis, which could be exploited for countermeasure development. Nevertheless, it is clear that any putative inferences on the roles of bacterial virulence mechanisms or phenotypes and host cell responses that are based on cell culture data must be examined in animal models to determine their possible relevance to infection.

## Supporting Information

S1 Fig Growth curves of 11 *B. pseudomallei* isolates. The growth curves, as determined by absorbance at OD_620_, showed no significant differences.
(TIF)

S2 Fig Infection of J774A.1 macrophages with *B*. *pseudomallei* strains 1106a or HBPUB10134a. **A.** The cells were infected in the presence or absence of cytochalasin D (CD) at MOIs of 16.3 (1106a, + CD), 31 (1106a,—CD), 32 (HBPUB10134a, + CD), or 28 (HBPUB10134a,—CD). The infected cells were incubated for 1 h, washed to remove nonphagocytosed bacteria, and incubated for 2 h in the presence of kanamycin. Shown are the mean viable counts recovered after the 1 h uptake period and after incubation with kanamycin (3 h). The number of HBPUB10134a (white bars) adherent to the macrophages (normalized as % of inoculum) was two-fold less than that of 1106a (black bars), as determined by the viable counts from cells infected 1 h with CD-treated inocula (*P* < 0.0001). The direct mean numbers of phagocytosed bacteria shown after washing and incubation (3 h,—CD) were not significant (*P* = 0.161). However, the extent of phagocytosis of HBPUB10134a was 11-fold less than that of 1106a when viable counts were normalized as a percentage of the inoculum; these values were 0.05% and 0.55%, respectively. The direct mean number of CFU recovered from—CD and kanamycin-treated 1106a-infected wells was significantly greater than that from HBPUB10134a-infected wells, *P* = 0.0353 (GraphPad t-test). These were the number of bacteria phagocytosed in the presence of CD (inset graph). **B**. The cells were infected at MOIs of 16.5 (1106a) or 19.2 (HBPUB10134a). The infected cells were incubated for 1 h, washed to remove unphagocytosed bacteria, and incubated for 2 h in medium with no antibiotic (3 h). Shown are the mean viable counts recovered after the 1 h uptake period and in either the medium recovered from the wells or from cell lysate after the 2 h incubation (3 h). At the 3 h time-point, the medium was first removed, and the cells were washed twice before being lysed to recover intracellular bacteria. Strain 1106a counts were greater than those of HBPUB10134a for the 1 h, 3 h lysate, and 3 h medium samples: *P* = 0.0047, *P* < 0.0001, and *P* = 0.0056, respectively. In addition, the viable counts recovered from the macrophage lysates at the 1 h and 3 h time points for 1106a were twice those of HBPUB10134a when normalized as a percentage of each strain’s cell inoculum.
(TIF)

S1 Table Brief summary of human clinical history of *B*. *pseudomallei* isolates. (DOCX)

S2 Table Phenotypes of macrophages infected with *B*. *pseudomallei* strains: Bacterial survival (DOCX)
