# Supplementary figures and images for: Characterization of Burkholderia pseudomallei Strains Using a Murine Intraperitoneal Infection Model and In Vitro Macrophage Assays
Source: PLoS One. 2015 Apr 24;10(4):e0124667. doi: 10.1371/journal.pone.0124667 (PMC4409376; doi:10.1371/journal.pone.0124667)

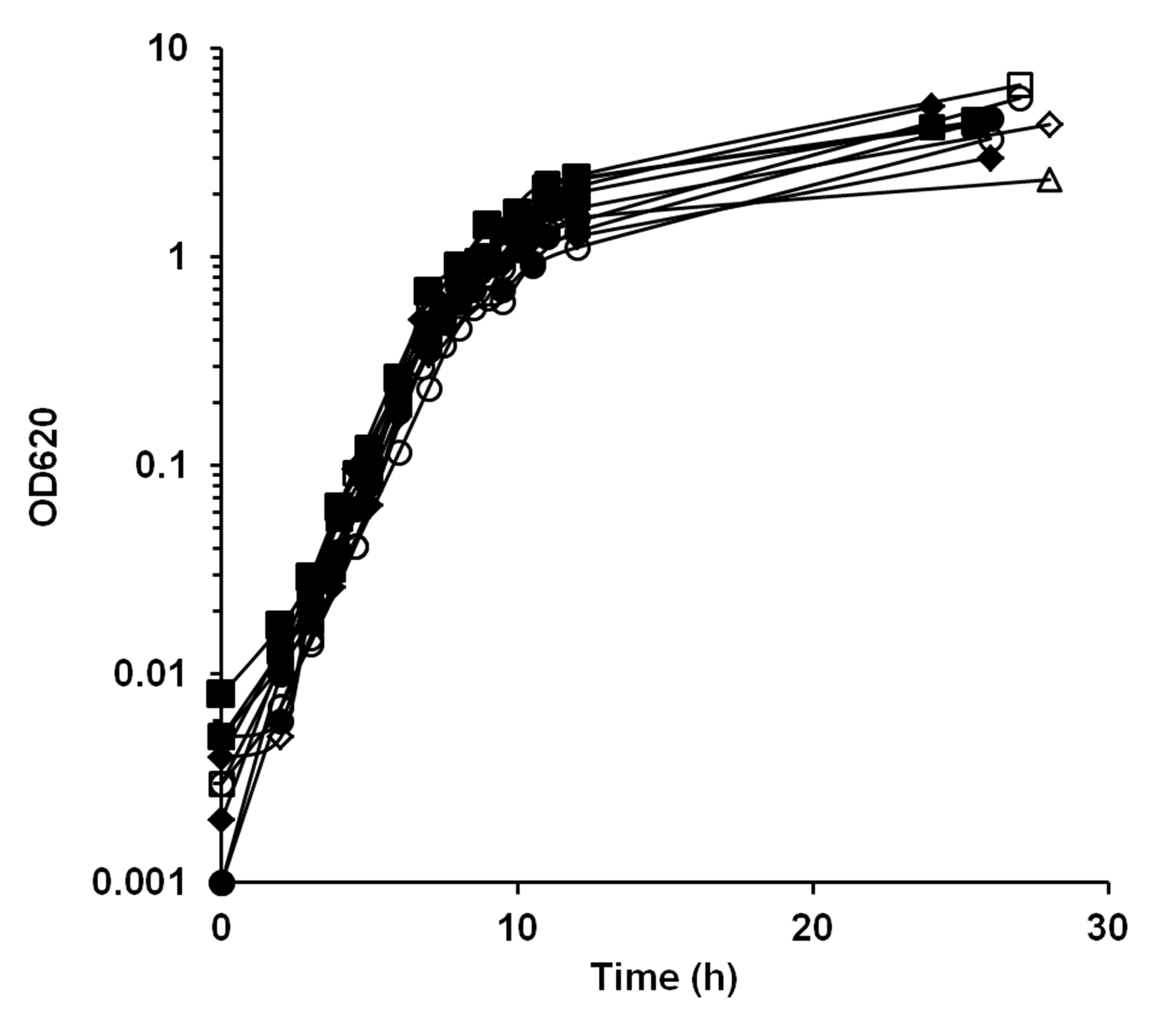

Supplement: S1 Fig — The growth curves, as determined by absorbance at OD620, showed no significant differences. (TIF) [file pone.0124667.s001.tif]

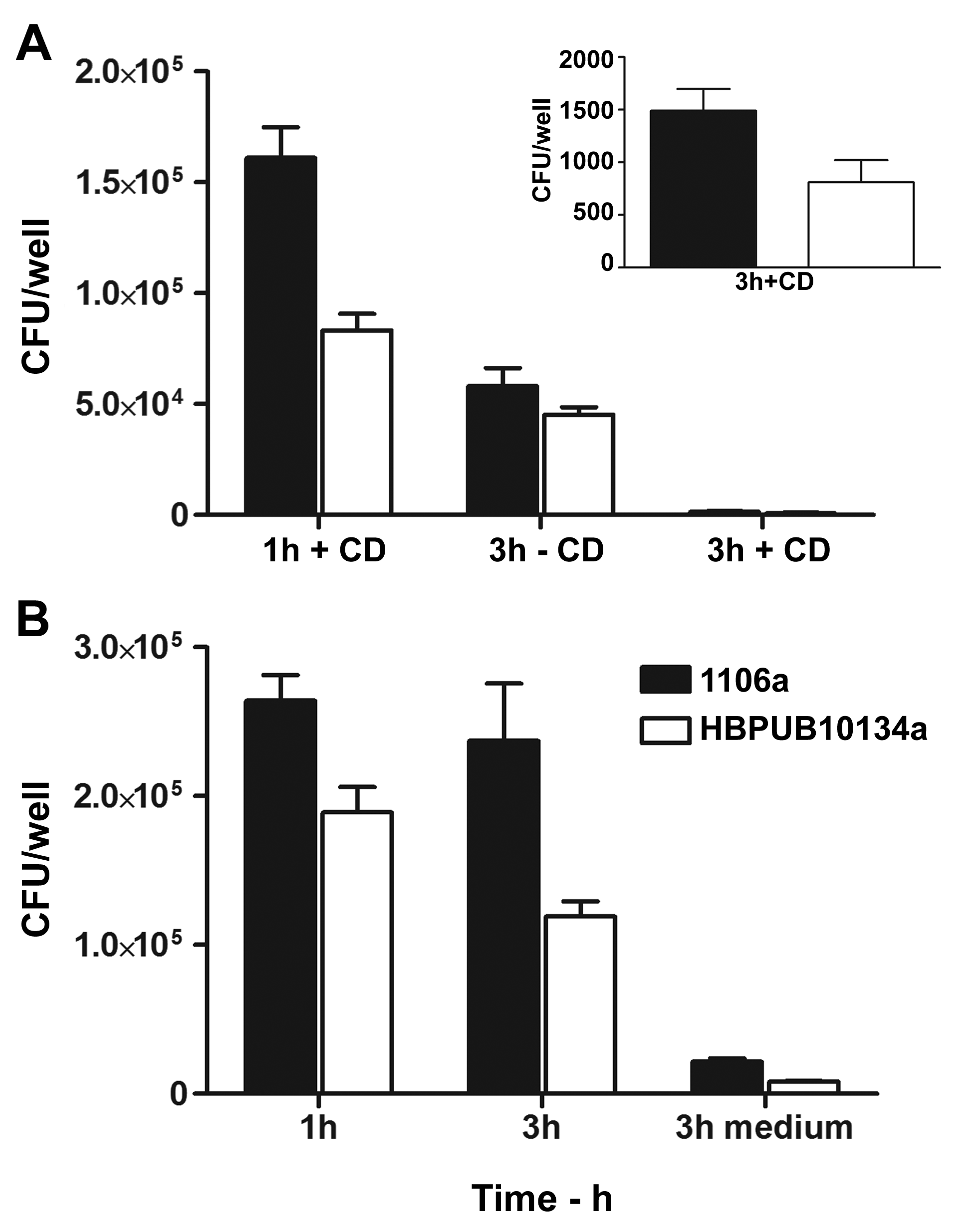

Supplement: S2 Fig — A. The cells were infected in the presence or absence of cytochalasin D (CD) at MOIs of 16.3 (1106a, + CD), 31 (1106a,—CD), 32 (HBPUB10134a, + CD), or 28 (HBPUB10134a,—CD). The infected cells were incubated for 1 h, washed to remove nonphagocytosed bacteria, and incubated for 2 h in the presence of kanamycin. Shown are the mean viable counts recovered after the 1 h uptake period and after incubation with kanamycin (3 h). The number of HBPUB10134a (white bars) adherent to the macrophages (normalized as % of inoculum) was two-fold less than that of 1106a (black bars), as determined by the viable counts from cells infected 1 h with CD-treated inocula (P < 0.0001). The direct mean numbers of phagocytosed bacteria shown after washing and incubation (3 h,—CD) were not significant (P = 0.161). However, the extent of phagocytosis of HBPUB10134a was 11-fold less than that of 1106a when viable counts were normalized as a percentage of the inoculum; these values were 0.05% and 0.55%, respectively. The direct mean number of CFU recovered from—CD and kanamycin-treated 1106a-infected wells was significantly greater than that from HBPUB10134a-infected wells, P = 0.0353 (GraphPad t-test). These were the number of bacteria phagocytosed in the presence of CD (inset graph). B. The cells were infected at MOIs of 16.5 (1106a) or 19.2 (HBPUB10134a). The infected cells were incubated for 1 h, washed to remove unphagocytosed bacteria, and incubated for 2 h in medium with no antibiotic (3 h). Shown are the mean viable counts recovered after the 1 h uptake period and in either the medium recovered from the wells or from cell lysate after the 2 h incubation (3 h). At the 3 h time-point, the medium was first removed, and the cells were washed twice before being lysed to recover intracellular bacteria. Strain 1106a counts were greater than those of HBPUB10134a for the 1 h, 3 h lysate, and 3 h medium samples: P = 0.0047, P < 0.0001, and P = 0.0056, respectively. In addition, the viable counts re [file pone.0124667.s002.tif]
