# Supplementary material for: Characterization of Burkholderia pseudomallei Strains Using a Murine Intraperitoneal Infection Model and In Vitro Macrophage Assays
Source: PLoS One. 2015 Apr 24;10(4):e0124667. doi: 10.1371/journal.pone.0124667 (PMC4409376; doi:10.1371/journal.pone.0124667)
Supplement: S1 Table — (DOCX) [file pone.0124667.s003.docx]

**Table S1. Brief summary of human clinical history of *B. pseudomallei* isolates.**

| **Strain** | **Origin** | **Collection Date** | **Brief Clinical History** |
| --- | --- | --- | --- |
| K96243 | Thailand | 1993 | sample from 34-year-old diabetic patient; short incubation,  rapid progression and death |
| 1026b | Thailand | 1993 | Blood sample from 29-year-old diabetic patient with non-fatal systemic disease |
| 1106a | Thailand | 1993 | sample from 23-year-old patient, pus from liver abscess |
| MSHR5858 | Australia | 2011 | sputum sample |
| 406e | Thailand | 1988 | toe swab from 21-year-old patient with systemic disease |
| MSHR5848 | Australia | 2011 | sputum sample |
| HBPUB10303a | Thailand | 2011 | tracheal suction sample |
| MSHR5855 | Australia | 2011 | sputum sample |
| MSHR305 | Australia | 1994 | autopsy sample from patient with fatal encephalomyelitis |
| MSHR668 | Australia | 1995 | Blood sample from 53-year-old patient with non-fatal systemic disease and encephalomyelitis |
| HBPUB10134a | Thailand | 2010 | tracheal suction sample |
