# Supplementary material for: Characterization of Burkholderia pseudomallei Strains Using a Murine Intraperitoneal Infection Model and In Vitro Macrophage Assays
Source: PLoS One. 2015 Apr 24;10(4):e0124667. doi: 10.1371/journal.pone.0124667 (PMC4409376; doi:10.1371/journal.pone.0124667)
Supplement: S2 Table — (DOCX) [file pone.0124667.s004.docx]

**Table S2.** **Phenotypes of macrophages infected with *B. pseudomallei* strains: Bacterial survival**

| **Table S2. Phenotypes of macrophages infected with *B. pseudomallei* strains: Bacterial survival** | | | | | | | | |
| --- | --- | --- | --- | --- | --- | --- | --- | --- |
|  | |  |  | **Adherence and phagocytosis:** | | **Recovery at 8h** | | |
| **Strain** | | **Panel^a^** | **MOI** | **Cell-associated (1h)  % inoculum** | **Phagocytosed (3h)  % inoculum** | **No. CFU/well** | **Compared to inoculum (%)** | **Compared to 3h counts (%)** |
| 1106a | | A | 17.2 | 2.3 | 1.1 | 5.69x10^5^ | 4.4 | 404 |
| HBPUB10134a | |  | 13.7 | 1.30 | 0.53 | 1.39x10^5^ | 1.35 | 252 |
| K96243 | | B | 27.5 | 2.1 | 0.5 | 9.78x10^4^ | 0.9 | 163 |
| HBPUB10134a | |  | 26.5 | 1.15 | 0.3 | 2.74x10^4^ | 0.26 | 87 |
| 1106a | | C | 5.8 | 10.7 | 5.4 | 9.00x10^5^ | 15.7 | 291 |
| MSHR668 | |  | 8.9 | 3.3 | 2.3 | 3.84x10^5^ | 4.3 | 190 |
| 1106a | | D | 12.4 | 3.75 | 3.0 | 1.34x10^6^ | 21.0 | 900 |
| MSHR5855 | |  | 7.7 | 2.5 | 1.9 | 5.57x10^5^ | 14.2 | 749 |
| 1026b | | E | 16.2 | 2.0 | 1.8 | 1.14x10^6^ | 7.5 | 420 |
| MSHR305 | |  | 16.9 | 2.35 | 1.5 | 7.33x10^5^ | 7.1 | 462 |
| 1106a | F | | 16.2 | 4.7 | 6.2 | 1.75x10^6b^ | 11.7^b^ | 187^b^ |
| 1026b |  |  | 16.2 | 2.0 | 1.8 | 1.14x10^6^ | 7.5 | 420 |
| ^a^Fig. 6, panels A - F.  ^b^Due to the large % cell loss, No. CFU/well recovered was likely significantly underestimated. | | | | | | | | |
